# Spectrum of rhodopsin mutations in Korean patients with retinitis pigmentosa

**Published:** 2011-04-01

**Authors:** Kwang Joong Kim, Cinoo Kim, Jeong Bok, Kyung-Seon Kim, Eun-Ju Lee, Sung Pyo Park, Hum Chung, Bok-Ghee Han, Hyung-Lae Kim, Kuchan Kimm, Hyeong Gon Yu, Jong-Young Lee

**Affiliations:** 1 Center for Genome Science, National Institute of Health, Chungcheongbuk-do, Korea; 2 Department of Ophthalmology, Seoul National University Hospital, Seoul National University College of Medicine, Seoul, Korea; 3 Department of Ophthalmology, Hallym University Medical School, Choncheon, Korea; 4 Department of Biochemistry, School of Medicine, Ewha Womans University, Seoul, Korea; 5 Department of Ophthalmology, Incheon Medical Center, Incheon, Korea

## Abstract

**Purpose:**

To determine the spectrum and frequency of rhodopsin gene (*RHO*) mutations in Korean patients with retinitis pigmentosa (RP) and to characterize genotype–phenotype correlations in patients with mutations.

**Methods:**

The *RHO* mutations were screened by direct sequencing, and mutation prevalence was measured in patients and controls. The impact of missense mutations to RP was predicted by segregation analysis, peptide sequence alignment, and in silico analysis. The severity of disease in patients with the missense mutations was compared by visual acuity, electroretinography, optical coherence tomography, and kinetic visual field testing.

**Results:**

Five heterozygous mutations were identified in six of 302 probands with RP, including a novel mutation (c.893C>A, p.A298D) and four known mutations (c.50C>T, p.T17M; c.533A>G, p.Y178C; c.888G>T, p.K296N; and c.1040C>T, p.P347L). The allele frequency of missense mutations was measured in 114 ethnically matched controls. p.A298D, newly identified in a sporadic patient, had never been found in controls and was predicted to be pathogenic. Among the patients with the missense mutations, we observed the most severe phenotype in patients with p.P347L, less severe phenotypes in patients with p.Y178C or p.A298D, and a relatively moderate phenotype in a patient with p.T17M.

**Conclusions:**

The results reveal the spectrum of *RHO* mutations in Korean RP patients and clinical features that vary according to mutations. Our findings will be useful for understanding these genetic spectra and the genotype–phenotype correlations and will therefore help with predicting disease prognosis and facilitating the development of gene therapy.

## Introduction

Retinitis pigmentosa (RP, OMIM 268000) is the most common type of inherited retinal degeneration and is characterized by progressive degeneration of the photoreceptors. RP is both clinically and genetically heterogeneous. Clinically, symptoms usually emerge as night blindness during adolescence, progress to legal blindness by middle age, and result in total vision loss in later life. Epidemiological studies consistently report a frequency of approximately 1 in 3,000–5,000 individuals, without apparent ethnic or racial distinctions [1]. However, clinical features, including age of onset and rate of progression, have been documented to vary according to genetic background [2]. Therefore, revealing the genotype–phenotype correlations for the prognosis of RP patients as well as identifying the causative mutations is of great importance.

Genetically, RP can be inherited as an autosomal dominant (adRP), autosomal recessive (arRP), or X-linked trait, and sporadic cases represent 40%–50% of all RP patients [3]. The genetic background of RP patients has not yet been elucidated owing to a large number of causative genes and variable inheritance patterns. Most genes for RP cause only a small proportion of cases, with the exception of the rhodopsin gene (*RHO*, OMIM 180380), which accounts for approximately 25% of adRP [4]. The first mutation of RP was discovered within *RHO* in 1990 [5], and since then more than 110 mutations have been found in different ethnic groups [6-10].

The mutation rate of the *RHO* gene appears to vary with ethnic background. The rate of *RHO* mutations is lower in the Japanese and Chinese population than in the United States [11,12]. The first gene defect found to cause RP, *RHO* p.Pro23His, is present in 12% of Caucasian American RP patients but occurs less frequently in other ethnic groups [11-13]. Although *RHO* is the most frequently reported adRP gene in many populations, there are no established data on *RHO* mutations in the Korean population. Such data would be of great utility toward the provision of effective genetic counseling and in predicting disease severity for RP patients.

Here, we present the results of a large-scale molecular screening of *RHO* mutations in Korean patients with RP. We describe and characterize the mutations identified in this study and discuss genotype–phenotype correlations.

## Methods

### Subjects

302 patients (183 males and 119 females, ages 7–86 years) with RP and their family members were recruited from the clinic for hereditary retina degenerations of the Department of Ophthalmology, Seoul National University Hospital (SNUH), Seoul, Korea, and the Korean Retinitis Pigmentosa Society, a nonprofit support network for Korean RP patients [14]. Informed consent was obtained from all participants before enrollment in the study, in accordance with the protocol approved by the Institutional Review Board at SNUH and at the Korea National Institute of Health. All protocols used in this study were also in full accordance with the tenets of the Declaration of Helsinki. Pedigrees of all patients were obtained through interviews. adRP was defined as RP showing direct vertical transmission in at least two generations with both males and females at equal risk and with no transmission to offspring of unaffected persons. It was considered as arRP when more than one member within the same sibship was affected and no other member of a previous generation was affected. Sporadic RP was diagnosed when there was no family history [15]. Among 302 patients included in this study, the most common inheritance pattern was sporadic RP (182, 60.3%), followed by arRP (55, 18.2%), adRP (38, 12.6%), and an unknown pattern (27, 8.9%). Patients with X-linked RP were excluded from the study. A total of 114 controls (48 males and 66 females, ages 34–70 years) were Korean individuals, recruited from SNUH or the Korea National Institute of Health, who had normal visual acuity and normal fundus photographs.

### Ophthalmic examinations

RP was diagnosed by retina specialists at SNUH when a patient showed bilateral retinal degeneration with typical pigmentation on funduscopic examination or rod dysfunction on a standard electroretinogram (ERG) with degenerative retina. Ophthalmic examination included the best-corrected visual acuity, refraction, intraocular pressure measurement, slit-lamp biomicroscopy, funduscopy, Goldmann perimetry, ERG, and optical coherence tomography (OCT; Cirrus HD-OCT, Carl Zeiss Meditec, Dublin, CA). Refractive errors were measured with an autorefractometer (ARK-7100; TOPKON, Tokyo, Japan), and Goldmann perimetry (Haag-Streit, Bern, Switzerland) with a V4e target was performed on all patients who were able to cooperate. ERG followed the guidelines of the International Society for Clinical Electrophysiology of Vision [16].

### Mutation screening

A total of 302 probands with RP were used for the mutation screening of *RHO* by direct sequencing. All variants identified in coding regions and splicing sites were further analyzed in 114 ethnically matched controls by sequencing. Genomic DNA was extracted from venous blood leukocytes using a FlexiGene DNA kit (Qiagen, Hilden, Germany) according to the manufacturer's instructions. Using the human rhodopsin gene sequence (NT_005612), PCR primers were designed to amplify the coding regions of five exons and flanking intronic regions. All primers and conditions are shown in Table 1. PCR products were purified and analyzed on an ABI 3730 DNA Analyzer (Applied Biosystems, Foster, CA). All detected mutations were sequenced bidirectionally at least twice for confirmation. The DNA mutation nomenclature is based on the cDNA sequence with the A of the translation initiation site corresponding to position +1.

**Table 1:** Primers.

**Target**	**Direction**	**Primer Sequence (5′→3′)**	**Amplicon**	**Tm (°C)**
Exon 1	Forward	TCAGAACCCAGAGTCATCCA	575 bp	58
	Reverse	GGACAGGAGAAGGGAGAAGG		
Exon 2	Forward	AAGCTCTCTCCTTCCCCAAG	496 bp	58
	Reverse	CCAGCCCTTGTAGCAACATT		
Exon 3–4	Forward	CATCCCCATCTGATCCATTC	815 bp	58
	Reverse	ACTGCTGACCCAAGACTGCT		
Exon 5	Forward	AGCTGGATTTGAGTGGATGG	788 bp	58
	Reverse	CCTACTGTGTGCCCCATTCT		

### Mutation analysis

Family segregation analysis of missense mutations was performed when patients from at least two generations are available in a family. Missense mutations were also evaluated for interspecies conservation using the ClustalX (ver. 2.0.12) multiple sequence alignment program [17]. Six computational algorithms, namely PolyPhen, PolyPhen-2, SIFT, PMut, SNPs3D, and PANTHER, were used to predict the functional impact of missense mutations identified in this study. The PolyPhen and PolyPhen-2 results for each variant were classified into three types: probably damaging, possibly damaging, and benign. We used HumVar-trained PolyPhen-2 for distinguishing mutations with drastic effects from other human variations, according to the recommendation by Adzhubei et al. [18]. Both probably damaging and possibly damaging mutations were classified as suspected pathogenic mutations. The output of SIFT showed a normalized probability score. Positions with normalized probabilities <0.05 were predicted to be deleterious, and those with normalized probabilities ≥0.05 were predicted to be tolerated. In this study, “affected protein function” was considered a suspected pathogenic mutation. PMut provides a simple answer with a reliability index: pathological and neutral. An output >0.5 is predicted to denote a pathological mutation, and an output <0.5 is neutral. In SNPs3D, two methods based on protein structure and amino acid conservation were used to assess the functional impact of nonsynonymous single nucleotide polymorphisms (SNPs). A positive support vector machine score indicates a variant classified as nondeleterious, and a negative score indicates a deleterious case. A higher score suggests more confident classification. Accuracy is significantly higher for scores greater than 0.5 or less than −0.5. PANTHER provides an online service for the prediction of functional effects of amino acid substitutions. The output score, the likelihood of the transition of one amino acid to another, is the negative logarithm of the probability ratio of the wild-type and mutant amino acids at a specific position. Lower scores indicate a higher probability of a deleterious functional effect.

## Results

### Mutation analysis

**Figure 1:**
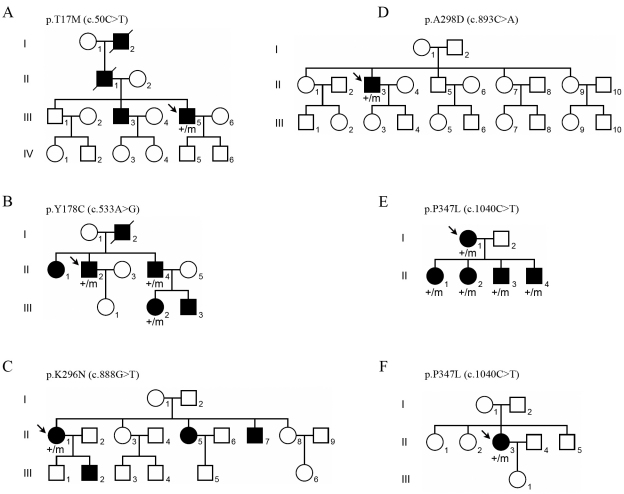
Pedigrees of families in which a rhodopsin mutation was identified. The mutation p.Y178C (**B**) and the mutation p.P347L (**E**) were segregated with disease phenotype. The newly identified mutation p.A298D (**D**) was found in a sporadic patient (II-3). Black and white symbols indicate affected and unaffected individuals, respectively. The mutation is represented by “m”; wild-type alleles are represented by “+”; arrows indicate probands.

A total of 302 probands from Korean families with RP were screened for mutations in *RHO.* By sequencing the exons and flanking intronic regions, five heterozygous mutations (c.50C>T, p.T17M; c.533A>G, p.Y178C; c.888G>T, p.K296N; c.893C>A, p.A298D; and c.1040C>T, p.P347L) were identified in six probands and were absent in 114 controls (Table 2). Of these, p.A298D has not been reported previously. The mutation frequencies were 10.5% (four mutation carriers in 38 patients, 4/38) for adRP, 1.1% (2/182) for sporadic RP, and 2.0% (6/302) in total. No mutation was detected in families with arRP and unknown patterns of inheritance. Mutations p.Y178C and p.P347L segregated with disease phenotype in studied families (Figure 1).

**Table 2:** Mutation screening of the *RHO* gene.

**Location**	**Nucleotide**	**Protein**	**Patient**	**Control**	**Note**	**Reference**
**Pathogenic**						
Exon 1	c.50C>T	p.Thr17Met	1/604	0/228	Reported	[30]
Exon 3	c.533A>G	p.Tyr178Cys	1/604	0/228	Reported	[9]
Exon 4	c.888G>T	p.Lys296Asn	1/604	0/228	Reported	[31]
Exon 4	c.893C>A	p.Ala298Asp	1/604	0/228	Novel	
Exon 5	c.1040C>T	p.Pro347Leu	2/604	0/228	Reported	[5]
**Benign**						
Intron 3	c.696+4C>T		28/604	11/228	Reported	rs56340615
Exon 4	c.891C>T	p.Ser297Ser	5/604	2/228	Reported	[31]
Intron 4	c.937–23G>A		172/604	58/228	Reported	rs2071092

Allele frequencies were measured in a total of 302 patients (604 chromosomes) and 114 controls (228 chromosomes).

**Figure 2:**
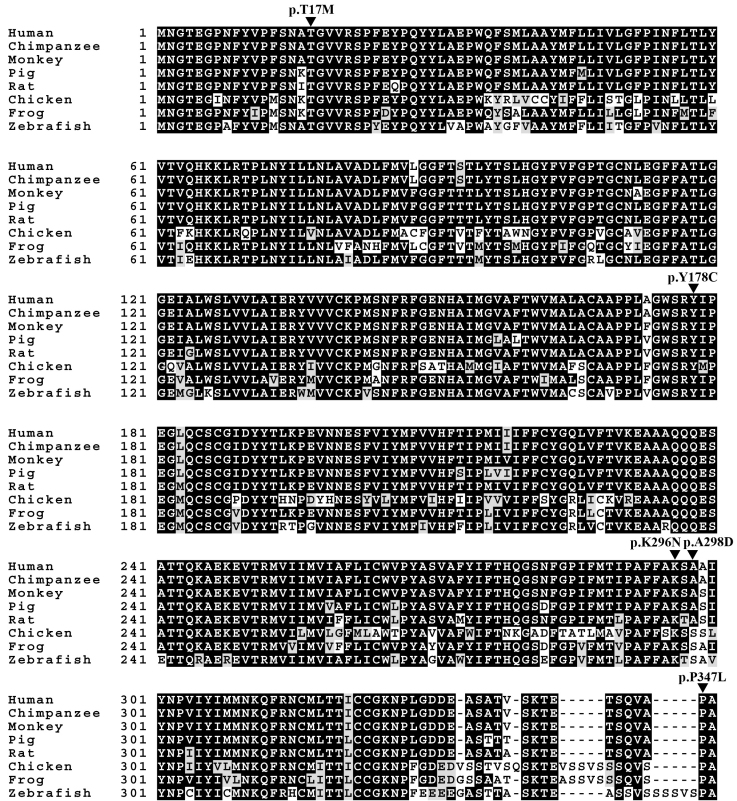
Conservation of rhodopsin protein. The positions of previously reported mutations (p.T17M, p.Y178C, p.K296N, and p.P347L) were highly conserved across species. However, the position of newly identified p.A298D mutation was conserved in mammals. Accession numbers of the protein sequences used for sequence comparison were as follows: chimpanzee, XP_516740.2; monkey, XP_001094250.1; pig, NP_999386.1; rat, NP_254276.1; chicken, NP_990821.1; frog, NP_001080517.1; zebrafish, NP_571159.1. The arrowhead shows the position of missense mutations identified in the present study.

The positions of previously reported mutations—p.T17M, p.Y178C, p.K296N, and p.P347L— were highly conserved across other species. However, in the case of the newly identified mutation, p.A298D, there were two residues, alanine and serine, across different species (Figure 2). To predict the impact on protein function of the missense mutations found in the present study, we performed an in silico analysis using six software packages. When more than half of the analytical results of the computational programs suggested that a mutation was pathological, the variant was classified as pathogenic. All five missense mutations, including the newly identified p.A298D, were considered to possibly affect protein function (Table 3). However, the confidence in the prediction for the effect of p.P347L was low, given that there was a gap at this position in many organisms.

**Table 3:** Prediction of the functional impact of missense mutations.

**Mutation**	**Phenotype**	**PolyPhen**	**PolyPhen2**	**SIFT**	**PMut**	**SNPs3D**	**PANTHER**
p.T17M	adRP	*Prob	**†Poss**	**‡A/0.00**	**§P/0.85**	**-0.56**	**-3.54**
p.Y178C	adRP	**Prob**	**Prob**	**A/0.00**	**P/0.71**	**-1.45**	**-4.37**
p.K296N	adRP	**Prob**	**Prob**	**A/0.00**	**P/0.72**	**-1.07**	na
p.A298D	#Sim	**Poss**	Benign	**A/0.00**	**P/0.92**	**-0.14**	−2.58
p.P347L	adRP, Sim	**Prob**	Benign	**A/0.00**	**P/0.95**	na	−1.92

*Prob, probably damaging; ^†^Poss, possibly damaging; ^‡^A, affect protein function; ^§^P, pathological; ^#^sim, simplex (sporadic) RP. na, not applicable. Bold means prediction of deleterious effect on protein structure or function.

#### p.Thr17Met

This mutation was detected as a heterozygote in one adRP proband (III-5 in Figure 1A), a 46-year-old male, and was not observed in 114 ethnically matched controls. The disease phenotype was transmitted from the affected father (II-1) to his two sons (III-3 and III-5). This position in the intradiscal region of rhodopsin is highly conserved among various organisms (Figure 2), and the substitution of threonine to methionine was predicted to affect protein function by six prediction software (Table 3).

#### p.Tyr178Cys

This mutation was observed as a heterozygous mutation in a proband of an adRP family (II-2 in Figure 1B) and was not found in controls. This mutant allele was shared between affected siblings (II-2 and II-4) and segregated with disease phenotype to an offspring (III-2). This position, located in an intradiscal region, is highly conserved (Figure 2), and the substitution was also predicted to be pathogenic by six prediction software (Table 3).

#### p.Lys296Asn

This mutation was detected as a heterozygote in one adRP patient (II-1 in Figure 1C), a 31-year-old female, and was not found in controls. The disease phenotype was shared among three of five siblings (II-1, II-5, and II-7) and was transmitted from the affected mother (II-1) to her son (III-2). The lysine at this position in the seventh transmembrane (TM) region of rhodopsin is highly conserved in all organisms tested (Figure 2). This substitution was predicted to be pathogenic by five prediction software (Table 3).

#### p.Ala298Asp

This mutation was newly identified in this study. This heterozygous mutation was observed only in a sporadic RP patient (II-3 in Figure 1D), a 55-year-old male, and was not observed in controls. The alanine at residue 298 in the seventh TM region is conserved in most mammals, but serine replaces alanine in chicken, frog, and zebrafish (Figure 2). This novel mutation was predicted to affect protein function by four prediction software (Table 3).

#### p.Pro347Leu

This mutation was detected as a heterozygous mutation in a proband (I-1 in Figure 1E) of an adRP family and was not observed in controls. In this family, the mutation segregated with disease from the affected mother (I-1) to all four children (II-1, II-2, II-3, and II-4). This mutation was also found in a sporadic RP proband (II-3 in Figure 1F), a 27-year-old female, as a heterozygote. The proline at residue 347 in the cytoplasmic region is highly conserved (Figure 2), and this mutation was predicted to be pathogenic by three prediction software (Table 3).

In the present study, three benign single nucleotide variants were also detected in patients with RP. One was a synonymous substitution at codon 297 (c.891C>T, p.S297S) and two were identified in splicing site (c.696+4C>T, intron 3; c.937–23G>A, intron 4). We also identified a novel missense substitution (c.895G>A, p.A299T) at codon 299 in a normal control. This nonsynonymous change was not detected in a patient with RP and was excluded from further mutational analysis. Except for p.A299T, there was no nucleotide variant found only in controls.

### Clinical evaluation in retinitis pigmentosa patients with missense mutation

The *RHO* mutations and phenotypes are summarized in Table 4. The patients ranged in age from 11 to 55 years. Most patients with missense mutations reported difficulty with night vision starting in the first decade or early second decade of life. Cataracts, especially the posterior subcapsular type, were evident in all patients older than 30 years of age, and both patients in their mid-forties (II-2 and II-4 in Figure 1B) exhibiting mutation p.Y178C had already undergone cataract surgery.

**Table 4:** The *RHO* mutations and phenotypes.

**Patient ID**	**Age/Sex**	**Mutation**	*Onset (y)	**BCVA: RE/LE (decimal)**	**Lens opacities**	**VF: RE/LE**	**ERG: Cone/Rod**	**OCT: RE/LE (CFT, μm)**
**III-5,****Figure 1A**	**45/M**	**p.T17M**	**14**	**0.6/0.5**	**NS**	**Central/5°**	**†na**	**na**
II-2, Figure 1B	47/M	p.Y178C	16	FC/NLP	IOL	Failure	Failure	202/188
**II-4,****Figure 1B**	**45/M**	**p.Y178C**	**12**	**0.15/0.4**	**IOL**	**na**	**Non-detectable**	**282/298**
III-2, Figure 1B	23/F	p.Y178C	13	0.8/0.6	Clear	na	na	207/213
**II-1,****Figure 1C**	**31/F**	**p.K296N**	**15**	**0.3/0.4**	**PSC**	**Central/7°**	**na**	**na**
II-3, Figure 1D	55/M	p.A298D	16	HM/LP	PSC	Failure	Non-detectable	231/267
**I-1,****Figure 1E**	**44/F**	**p.P347L**	**Teens**	**LP/LP**	**NS**	**Failure**	**Non-detectable**	**161/152**
II-1, Figure 1E	17/F	p.P347L	15	1.0/0.7	Clear	Peripheral constriction	Reduction/Non-detectable	387/560
**II-2,****Figure 1E**	**15/F**	**p.P347L**	**15**	**0.8/0.8**	**Clear**	**Normal**	**Normal range/Non-detectable**	**376/428**
II-3, Figure 1E	13/M	p.P347L	12	1.2/1.0	Clear	Normal	Normal range/Non-detectable	295/291
**II-4,****Figure 1E**	**11/M**	**p.P347L**	**Not yet**	**0.7/0.5**	**Clear**	**Normal**	**Normal range/Reduction**	**400/499**
II-3, Figure 1F	26/F	p.P347L	23	0.6/0.7	PSC	Central/10°	Non-detectable	216/175

BCVA, best corrected visual acuity; CFT, central foveal thickness; ERG, electroretinogram; FC, finger count at specified distance; HM, hand motion; IOL, intraocular lens; LE, left eye; LP, light perception; NLP, no light perception; NS, nuclear sclerosis; OCT, optical coherence tomography; PSC, posterior subcapsular opacity; RE, right eye; VF, visual field. *Onset (y), age at onset of night blindness. ^†^na, data not available.

**Figure 3:**
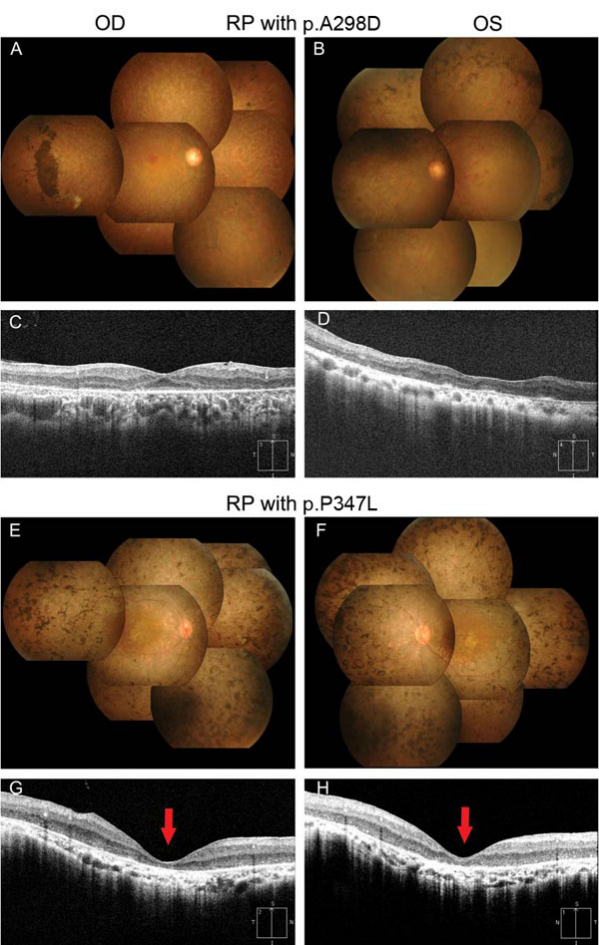
Comparison of fundus photographs and spectral domain optical coherence tomography (SD-OCT) between patients with the p.A298D and the p.P347L mutations. Fundus photographs (**A**, **B**, **E**, **F**) show typical retinitis pigmentosa (RP) features: retinal degeneration with pigmentation, atrophy of retinal pigment epithelium (RPE), and attenuated vessels, which involved the area inside the vascular arcade. Compared with a 55-year-old patient with the p.A298D mutation (**A**, **B**), a 44-year-old patient with the p.P347L mutation (**E**, **F**) had more severe retinal pigmentation, despite being 11 years younger. SD-OCT (**C**, **D**, **G**, **H**) revealed the degeneration of photoreceptor and RPE layers and the disruption of the inner and outer segment junction of the photoreceptor in both patients. In particular, severe foveal atrophy in the patient with the p.P347L mutation resulted in a large decrease of the central foveal thickness: 161 μm in the right eye (**G; arrow**) and 152 μm in the left eye (**H**; arrow).

Patients older than 40 years of age with the p.A298D or p.P347L mutation showed poor visual acuity of light perception or no light perception in at least one eye. For one of two patients older than 40 years of age with the p.Y178C mutation, visual acuity was no light perception and finger count in each eye. However, the patient with mutation p.T17M (III-5 in Figure 1A) maintained much better visual acuity (20/40) despite being a similar age. The fundus examination revealed intraretinal bone-spicule pigment deposits or retinal degeneration in all patients, except the youngest who was aged 11 (II-4 in Figure 1E). The extent of retinal pigment epithelial atrophy, the number of retinal pigment deposits, and the attenuation of the retinal arteries were greater in the older individuals. Retinal pigmentary degeneration was most severe in a patient with mutation p.P347L (I-1 in Figure 1E) compared with patients of similar age and with other mutations (Figure 3).

All patients older than 20 years had a severely constricted visual field, with less than the central 10° of radius remaining, even in a 26-year-old patient with mutation p.P347L (II-3 in Figure 1F). An ERG was extinguished or failed in older patients because of their advanced stage or poor visual acuity. In comparison with the four affected children in an adRP family with mutation p.P347L, one 17-year-old patient (II-1) showed peripheral constriction of the visual field and decreased amplitude and prolonged implicit time in a cone ERG, whereas patients aged 15, 13, and 11 (II-2, II-3, and II-4) showed unremarkable findings in these tests (Figure 1E). However, the rod response in the ERG was extinguished or decreased in all children. When we compared OCT findings between a p.P347L patient (I-1 in Figure 2E) with a p.A298D patient (II-3 in Figure 1D), severe decreases in central foveal thickness were observed in both eyes of the p.P347L patient, resulting in advanced foveal atrophy (Figure 3C,D,G,H). Interestingly, cystoid macular edema was detected by spectral domain OCT in both eyes of all four children with mutation p.P347L.

## Discussion

As a result of the founder effect, mutations can occur frequently in one population yet be rare or absent in another. For example, the p.Pro23His mutation descends from a common white ancestor and has not been reported in other ethnic groups [19], including our present study. In this study, we screened *RHO* mutations in Korean RP patients and found five missense mutations, including a novel one. These missense mutations related to RP phenotype and were not identified in ethically matched controls. The proportion of *RHO* mutations that related to RP in Korean patients was approximately 2.0% (6/302), which is lower than that in the United States [9], and Europe [13] but similar to that in other Asian populations, such as Japanese (5.9%) [20], Chinese (2.0%–5.6%) [11,21], and Indian (2.0%) [6], suggesting an ethnicity-dependent mutation rate.

The *RHO* gene mainly causes adRP, except several mutations that have been reported in the arRP family [22]. Generally, autosomal recessive inheritance has been assumed to be the major cause of sporadic RP. Therefore, the finding of heterozygous mutations of *RHO* (p.A298D and p.P347L) in sporadic patients (II-3 in Figure 1D and II-3 in Figure 1F) was unexpected. In the case of no segregation pattern among family members, it is difficult to predict whether a newly detected mutation is pathogenic, particularly when it is a missense substitution. For the p.A298D mutation, which was newly identified in a sporadic patient, we considered three aspects for the evaluation of pathogenicity. First, we surveyed whether the same mutation was found in normal controls. Second, we analyzed the amino acid conservation among different species. Third, we assessed whether the alteration is suggestive of protein malfunction by in silico analysis. The p.A298D mutation was not observed in a panel of 114 normal controls (228 chromosomes). In addition, the alanine residue at codon 298 was relatively well conserved in mammals. Notably, four different algorithms gave fairly consistent predictions of pathogenicity for the p.A298D substitution (Table 3). Although segregation analysis was not applicable, these results implied that the newly identified mutation p.A298D may affect RHO protein structure and/or function, thus resulting in RP.

The substitution of c.696+4C>T in the splicing donor site of intron 3 and the substitution of c.937–23G>A in the branch site of intron 4 were not considered to be related to RP by affecting pre-mRNA splicing because there were no significant differences in allele frequencies between patients and controls. Interestingly, two of five missense mutations and one synonymous change were found in the seventh TM helix of rhodopsin. Together with p.P347L, the most common *RHO* mutation causing RP worldwide, these positions between codon 296 and codon 298 are thought to be a cluster of mutation “hot spots,” at least in the Korean population.

The phenotype–genotype correlation in RP patients with the missense mutations is also worth noting. The mode of inheritance is believed to play an important role in determining the prognosis of the disease. In general, adRP has the slowest progression, while X-linked RP tends to induce the most severe form of the disease [23,24]. Furthermore, even within the different mutations in the same gene causing adRP, extensive phenotypic variations have been reported. For example, *RHO*-related RP from mutations involving codon 347 produces a more severe phenotype than RP involving codon 23 [25]. Specifically, vision loss was estimated to occur approximately 17 years earlier in p.P347L patients compared with p.P23H patients [26]. We reconfirmed that Korean RP patients carrying the p.P347L mutation experienced severe clinical features, as has been previously reported for other ethnic groups [11,26-28]. Also, we reconfirmed the mild phenotype of the p.T17M mutation in the Korean RP patients, which has been previously reported as a mutation with less impaired rod and cone function in other ethnic group [29].

However, phenotypes of patients with the p.Y178C mutations as well as the novel mutation p.A298D have not been reported elsewhere. When comparing clinical features after correcting for age, patients with the p.Y178C, p.A298D, and p.P347L mutations exhibited worse visual acuity, while a patient with mutation p.T17M maintained better visual acuity. When we compared a p.A298D patient with a p.P347L patient for fundus and OCT findings, pigmentary degeneration and foveal atrophy were more severe in the p.P347L patient (Figure 3). Therefore, in our study phenotypes of patients with the *RHO* mutations were most severe in p.P347L patients, less severe in p.Y198C and p.A298D patients, and relatively moderate in a p.T17M patient. However, it was not possible to statistically assess phenotypic differences among our mutations because there was small number of patients per each missense mutation, with the exception of mutation p.P347L. A more accurate description of disease severity associated with these mutations would require a longitudinal follow-up of large patient cohorts.

In conclusion, we revealed the spectrum and frequency of *RHO* mutations in Korean RP patients. *RHO* mutations were found in 2.0% of Korean patients and present in sporadic RP as well as in adRP families. Moreover, the clinical features varied according to the mutation. The newly identified p.A298D mutation showed pathological characteristics in genetic and phenotypic analyses. Analysis of additional families or identification of a biochemical defect in the mutated rhodopsin is needed to confirm the role of this allele in producing RP. This study is the largest screening of *RHO* mutations in a Korean population. Understanding the unique patterns of frequent mutations in a specific ethnic group may facilitate the development of molecular diagnosis and personalized gene therapy for RP.
